# Extracellular Vesicles: Recent Developments in Aging and Reproductive Diseases

**DOI:** 10.3389/fcell.2020.577084

**Published:** 2020-09-17

**Authors:** Yu Liu, Qiuzi Shen, Ling Zhang, Wenpei Xiang

**Affiliations:** Institute of Reproductive Health and Center for Reproductive Medicine, Tongji Medical College, Huazhong University of Science and Technology, Wuhan, China

**Keywords:** extracellular vesicles, isolation, aging, polycystic ovary syndrome, endometriosis

## Abstract

Extracellular vesicles (EVs), present in cell culture media and several body fluids, play a prominent role in intercellular communication under physiological and pathological conditions. We performed a systematic literature search to review evidence regarding the existence, composition, and release of different EVs, as well as the biomarkers, cargos, and separation methods. We also reviewed the potential of EVs to transport cargos and alter the function and phenotype of recipient cells associated with aging and reproductive diseases, including polycystic ovary syndrome and endometriosis. In aging, EVs promote inflammatory reactions and offsetting the occurrence of aging. In the polycystic ovary syndrome and endometriosis, EVs and their cargos are involved in the occurrence of diseases, therapeutic strategies, and perform as non-invasive biomarkers. As the study of EVs is still in the early stages, it is not surprising that most of the current literature only describes their possible roles.

## Introduction

Intercellular communication has been shown to play an essential role in diverse physiological processes, including cell proliferation, development, and differentiation. Published literature in recent years has revealed a new mechanism of intercellular communication, namely via the release of extracellular vesicles (EVs). Classically, intercellular communication includes endocrine, paracrine, and autocrine or intercellular gap junctions, a kind of direct cell-cell contact, and secreted some factors. In the group of secreted factors, we will focus here on the roles of EVs. EVs secreted outside of the cell can serve as vehicles for the transport of cargo to recipient cells (Raposo and Stoorvogel, 2013). EVs display a diverse range of sizes and are present in cell culture media (under both normal and pathological conditions) and several body fluids (Caby et al., 2005; Admyre et al., 2007; Ogawa et al., 2008; Gonzales et al., 2009). The cargo of EVs contains biologically active molecules, such as nucleic acids (DNA, RNA, microRNAs and long non-coding RNAs), proteins, lipids, and nicotinamide phosphoribosyltransferase (eNAMPT) (Yoshida et al., 2019).

EVs have been shown to be involved in numerous biological functions and pathological processes (Baek et al., 2016). Meanwhile, EVs and their cargos act as non-invasive markers of various diseases (Li et al., 2009; Mitchell et al., 2009; Choi et al., 2011; Saman et al., 2012; Ostergaard et al., 2013; Jakobsen et al., 2015). Abnormal EV levels may be one of the causes of aging and reproductive diseases (including polycystic ovary syndrome (PCOS) and endometriosis), and are closely related to the occurrence, development, and prognosis of these diseases. Moreover, EVs can alleviate aging phenotypes, and promote cell proliferation (Liu et al., 2019). Such developments offer new therapeutic strategies for the treatment of PCOS in the future. The purpose of this review is to describe the present knowledge of the role of EVs as cell-to-cell messengers in aging and reproductive diseases.

## Methods

**FIGURE 1:**
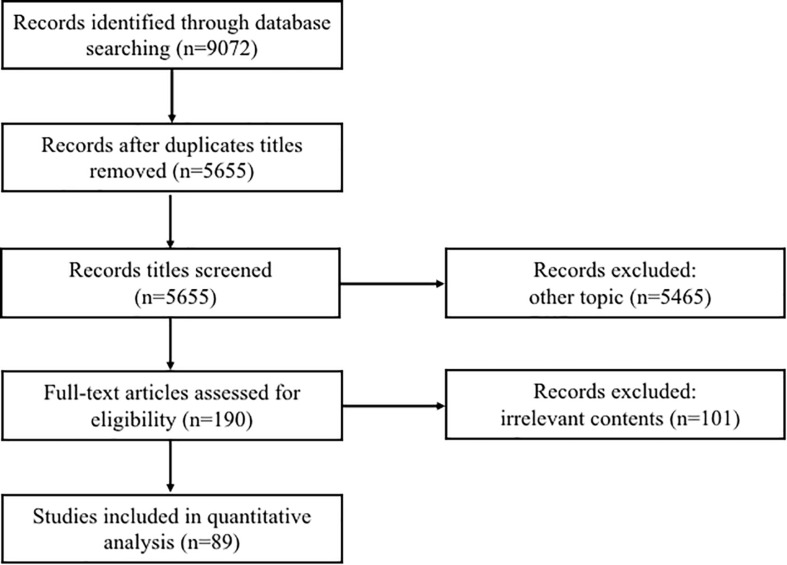
Schematic of study selection.

For this review, including three strategies: literature search, study selection, and results summary. We conducted a systematic online literature search of the PubMed and Web of Science databases and searched all published articles since the database’s creation to 2019. We used the following query: (‘extracellular vesicles’ or ‘microvesicles’ or ‘microparticles’ or ‘exosomes’) and (‘comparison of isolation method’ or ‘aging’ or ‘polycystic ovary syndrome’ or ‘endometriosis’). Both animal and human studies were considered suitable for this review. Additionally, all relevant studies were identified and included. These types of EVs do not include “apoptotic bodies” and “apoptotic vesicles”. Any duplicate articles were eliminated. After screening the title and/or abstracts, if the article was found to be unrelated to the study, was excluded. A total of 9072 records were retrieved from the two databases. After removing duplicates titles and other topic articles, the full-text articles of 190 articles were reviewed and 89 were considered relevant and included in this review (Figure 1).

## Extracellular Vesicles-Identification

**FIGURE 2:**
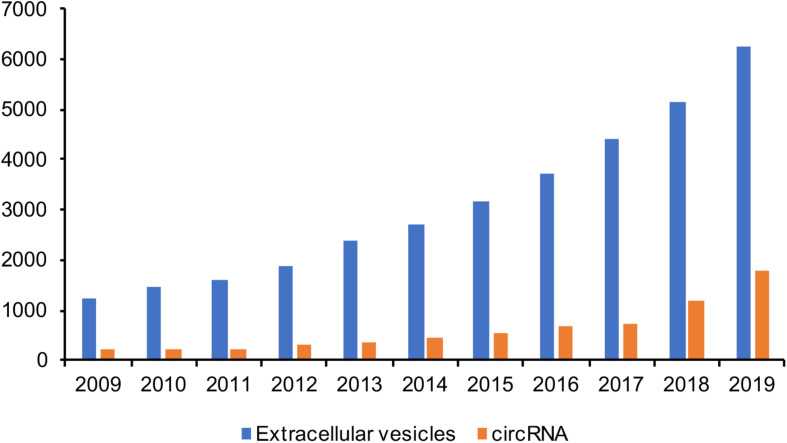
Number of papers published in EVs research in the last decade.

Over the past ten years, the number of published papers on EVs has increased exponentially, which is in sharp contrast with the field of circRNA. This also proves that researchers have a growing understanding of EVs, as seen in Figure 2, to the extent that the 2013 Nobel Prize in Physiology or Medicine was awarded to three scientists (James E. Rothman, Randy W. Schekman, and Thomas C. Südhof) working on the transport system of vesicles inside cells. In fact, the study of EVs dates back even further. It was in 1982 when EVs were first reported in seminal plasma (Stegmayr and Ronquist, 1982). EVs were initially considered as “cellular garbage”, although they are now known as important mediators of intercellular communication, participating in and activating a variety of cell signaling pathways (Raposo and Stoorvogel, 2013).

**FIGURE 3:**
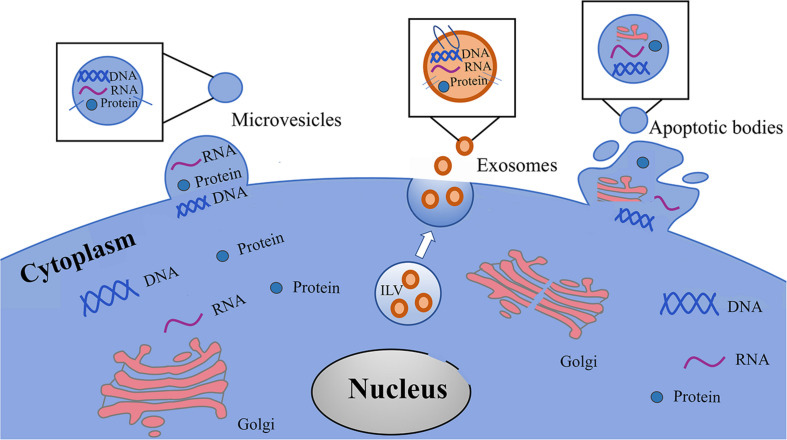
Types of EVs in the cells culture media and body fluids. EVs are classified in three heterogeneous population, including exosomes, microvesicles and apoptotic bodies. Exosomes are originated from intraluminal vesicles (ILVs) reside within multivesicular endosomes (MVEs) that are the precursor of exosomes. Microvesicles shedding from the plasma membrane and subsequently released into the extracellular space. Apoptotic bodies are released from dying cells.

EVs are a heterogeneous population (Bobrie et al., 2012; Raposo and Stoorvogel, 2013; Kowal et al., 2016), and can be classified into three categories according to their biogenetic pathway and physical characteristics: exosomes, microvesicles (MVs), and apoptotic bodies (Figure 3 and Table 1). Exosomes are small vesicles with lipid bilayers that contain cytoplasmic components of secretory cells which indirectly reflect the state and nature of the secretory cells and are biologically equivalent to the cytoplasm of the lipid bilayer. The external domains of transmembrane proteins are exposed in the extracellular space (Schorey et al., 2015). Exosomes are derived from intraluminal vesicles (ILVs) residing within multivesicular endosomes (MVEs) (van Niel et al., 2018). MVs can also be called microparticles (MPs), formerly known as ‘dust’, and are shed from the plasma membrane and subsequently released into the extracellular space (Mathieu et al., 2019). MVs may be released faster than exosomes, which is directly related to their production mechanism. Apoptotic bodies are released from dying cells (Colombo et al., 2014; Yanez-Mo et al., 2015). The production of EVs has been widely observed in bacteria, humans, and plants (Deatherage and Cookson, 2012; Schorey et al., 2015; Robinson et al., 2016), and demonstrates an evolutionarily conserved intercellular signaling mechanism. Exosomes are small vesicles of 40–150 nm in diameter (Jeppesen et al., 2019), although can potentially reach as large as 200 nm (Colombo et al., 2013), and therefore overlap the range of virus sizes (Gyorgy et al., 2011). MVs vary in size from 100 to 1,000 nm in diameter (Marques et al., 2013), up to 10 μm, and overlap the range of bacteria sizes (Akers et al., 2013). Apoptotic bodies are the largest vesicles (>1,000 nm) of the three classes and overlap the rang of platelet diameter (Hristov et al., 2004; Atkin-Smith et al., 2015). In fact, the diameter of vesicles does not sufficiently differentiate exosomes and MVs because in some instances their diameters may overlap with each other. Typical biomarkers for exosomes include transmembrane tetrad proteins (e.g., CD9, CD63, and CD81), Alix, TSG101, Flotillin-1, HSC70, and syntenin-1 (Mathivanan et al., 2010; Colombo et al., 2014). Discriminatory markers of MVs include annexin A1, ARF6, KIF, RACGAP, exportin-2, and chromosome segregation 1-like protein (Muralidharan-Chari et al., 2009; Xu et al., 2016; Tricarico et al., 2017; Jeppesen et al., 2019). Phosphatidylserine (PS), thrombospondin, C3b complement protein, calreticulin, and VDAC1 have been proven to be useful markers for apoptotic bodies (Takizawa et al., 1996; Fadok et al., 2001; Friedl et al., 2002; Jeppesen et al., 2014; Van Deun et al., 2014).

**TABLE 1:** Summary of key characteristics of microvesicles, exosomes and apoptotic bodies.

	Microvesicles	Exosomes	Apoptotic bodies	References
Size (nm)	100 to 1,000/10,000 overlaps with bacteria size	40 to 150/200 overlap with virus size	>1,000 overlaps with platelet diameter	Hristov et al., 2004; Gyorgy et al., 2011; Akers et al., 2013; Colombo et al., 2013; Marques et al., 2013; Atkin-Smith et al., 2015; Jeppesen et al., 2019
Isolation procedure	Sequential centrifugation (300g/2,000g/10,000g)	Ultracentrifugation (UC), commercial kits, immunoaffinity, size exclusion, polymeric precipitation, microfluidics,	Centrifugation(300g), sequential filtering (5 and 1μm filters) and centrifugation(2,000g)	Chen et al., 2010, Hartman et al., 2011, Taylor et al., 2011; da Silveira et al., 2012; Sohel et al., 2013; Witwer et al., 2013; Boing et al., 2014; Van Deun et al., 2014; Greening et al., 2015; Xu et al., 2015
Selected protein markers	Annexin A1, ARF6, KIF, RACGAP, exportin-2 and chromosome segregation 1–like protein	Transmembrane tetrad proteins (including CD9, CD63, and CD81), Alix, TSG101, Flotillin-1, HSC70 and syntenin-1	VDAC1, PS, thrombospondin, C3b, calreticulin.	Takizawa et al., 1996; Fadok et al., 2001; Friedl et al., 2002; Muralidharan-Chari et al., 2009; Mathivanan et al., 2010; Colombo et al., 2014; Jeppesen et al., 2014, 2019; Van Deun et al., 2014; Xu et al., 2016; Tricarico et al., 2017
Biogenesis	Shedding from the plasma membrane and subsequently released into the extracellular space	Originate from intraluminal vesicles (ILVs) reside within multivesicular endosomes (MVEs) and are the precursor of exosomes	Released from dying cells	Colombo et al., 2014; Yanez-Mo et al., 2015; van Niel et al., 2018; Mathieu et al., 2019

## Extracellular Vesicles Isolation Methods

Many techniques have been established to isolate different EVs. Ultracentrifugation (UC) is one of the most commonly used methods for exosomal isolation. Extension of UC time increases the degree of pollution of non-vesicular particles (Cvjetkovic et al., 2014). Non-vesicular components are likely to be contaminating and mainly include high molecular weight proteins and protein aggregates that co-precipitate with exosomes during the UC process (Tauro et al., 2012). Moreover, UC shows the lowest recovery, but the highest protein purity of all exosomal isolation methods (Tang et al., 2017). Therefore, the UC method is the ideal option for proteomic analysis of exosomes (Alvarez et al., 2012; Rekker et al., 2014). In recent years, a series of commercial kits have been developed for the isolation and extraction of exosomes (Hartman et al., 2011; da Silveira et al., 2012; Sohel et al., 2013). Compared to the UC method, commercial kits have simple methods (Helwa et al., 2017), often produce a significantly higher yield of exosomes (Helwa et al., 2017), and show a higher extraction efficiency (Tang et al., 2017). However, albumin impurities in the final extract are common. Notably, commercial kits produced the highest numbers of exosome miRNA and mRNA. Therefore, the use of commercial kits is an ideal method for subsequent RNA profiling analysis (Alvarez et al., 2012). Lastly, immunoaffinity capture is another method for separating and purifying exosomes. This method allows for the purification of high levels of exosomes and the exosome-associated proteins (Greening et al., 2015), and this is suitable for protein analysis of exosomes.

The selection of exosome isolation methods will have a substantial impact on the following RNA and protein analysis. Therefore, an appropriate exosome isolation method is essential to reveal exosomal-specific contents, and biological functions.

## Extracellular Vesicles and Aging

Aging can be defined by a decline in multiple biological functions. There are many aging-associated disorders and chronic diseases, for example, sarcopenia (Landi et al., 2018), cancers, and cardiovascular diseases. This time-dependent process is characterized by the accumulation of cellular damage, widely recognized as a common cause of aging (Kirkwood, 2005; Gems and Partridge, 2013). A typical hallmark of senescent cells is the stability of permanent cell cycle arrest. Generally, the DNA synthesizing ability of senescent cells are typical of the G1 phase (Di Leonardo et al., 1994; Herbig et al., 2004). Aging has nine hallmarks: genomic instability, telomere shortening, epigenetic alterations, loss of proteostasis, deregulated nutrient sensing, mitochondrial dysfunction, senescence, stem cell exhaustion, and alteration in intercellular communication (Shiels et al., 2017). Cells in senescence exhibit a senescence-associated secretory phenotype (SASP), triggering the loss of DNA replication capacity (Campisi and d’Adda di Fagagna, 2007). Previous studies about aging have focused on traditional fields such as genetic alterations and epigenetics. With the development of EVs separation technology and the deepening of theoretical research, we have a new understanding of the mechanism of aging. Recently, EVs and their contents have received extensive attention in the research of aging mechanism.

EVs play a fundamental role in aging (Pusic and Kraig, 2014; Alique et al., 2017). Takasugi et al. (2017) demonstrated that EVs secreted by DNA-damaging agent doxorubicin induced senescent RPE-1 cells are important mediators of the pro-tumorigenic function of senescent cells. EVs-associated EphA2 secreted from senescent cells binds to ephrin-A1, which is highly expressed in several types of cancer cells and promotes cell proliferation through EphA2/ephrin-A1 reverse signaling. However, recent finding indicated that induced pluripotent stem cells (iPSCs) produce a large number of EVs, which could alleviate the aging-associated phenotypes of senescent mesenchymal stem cells (MSCs), promote cell proliferation, and mitigate progerin-induced senescence in premature aging cell models (Liu et al., 2019). Research into stem cells and their secreted EVs could help address the negative effects of senescent cells. This research would differ in that it requires the use of different cell sources of EVs.

At present, there are many controversies about whether EVs secretion in senescent cells is increased or decreased. In 2008, Lehmann et al. (2008) first certified that the release of EVs secretion increased during proliferative senescence in normal human diploid fibroblasts. This increase is regulated by p53 and TSAP6 (the target gene of p53). However, the exact mechanism of this regulation remains unclear. However, Eitan et al. (2017) provided an analysis of EVs in peripheral blood circulation through an epidemiologic and longitudinal study, and showed that the concentration of EVs significantly decreased with increasing age. This contrasting age-related decrease in EVs may be partly attributed to the fact that EVs from senescent individuals are easier to internalize than those from more young and healthy individuals (Eitan et al., 2017). The protein levels in EVs may be account for the age-related concentration changes and internalization activation. Recent studies revealed that the apoptosis markers such as p53, cleaved PARP, and cleaved Caspase-3 are significantly decreased, while proteins including CD151 and tetraspanin are markedly increased with cell senescence (Eitan et al., 2017). Therefore, the change of EVs levels in senescent cells needs further study.

The contents of EVs are also closely related to senescence, such as miRNA, inflammatory cargos, nicotinamide phosphoribosyltransferase, DNA and C24:1 ceramide.

## miRNA

Recently, a new mechanism of intercellular communication mediated by exosome-associated miRNAs has attracted widespread attention (Valadi et al., 2007). miRNAs are short non-coding RNA (ncRNA) molecules that can act as gene regulators by inhibiting translation or binding to the three primes’ untranslated region (3’-UTR) of target messenger RNA (mRNA) to induce degradation of the target mRNA transcripts (Gasparello et al., 2019). miRNAs are therefore believed to be important in a range of physiological processes and the regulation of the development of many diseases. Moreover, Wei et al. (2017) recently analyzed extracellular RNA (exRNA) of EVs secreted by glioblastoma cells *in vitro* and found that ncRNA composed the majority of exRNA, instead of mRNA. This indicates that miRNAs play a significant and ever-growing role in the implementation of EVs function.

Studies have shown that exosome miRNA can be transported to surrounding tissues or cells and exert either a positive or negative impact, as summarized in Table 2. Several exosome-associated miRNAs are important regulators of senescence and cellular senescence. Exosomal miRNAs from senescent cells can be transported to surrounding cells and lead to aging (Smith-Vikos and Slack, 2012; Urbanelli et al., 2016). For example, Davis et al. (2017) treated bone marrow mesenchymal stem cells (BMSCs) of young mice with EVs derived from the bone marrow of aging mice and found that osteogenic differentiation was inhibited and BMSCs senescence was induced. This phenomenon can be mimicked by the transfection of miR-183-5p into BMSCs. Aging of the brain is associated with the loss of myelin, which has been shown to directly cause cognitive decline (Pusic and Kraig, 2014). In this study, serum-derived exosome miRNA in young Wistar rats promoted the differentiation of primary oligodendrocyte precursor cells (OPC) and improved the ability of remyelination in older Wistar rats. In the microenvironment of bone marrow, age-related miRNA changes can inhibit bone formation and promote bone resorption, leading to the osteoporosis. Exosomes derived from older rats BMSCs promoted the occurrence of osteoporosis and had higher levels of miR-31a-5p, compared to that of younger rats. Therefore, exosome miRNA is considered to be as an important mediator in the age-related bone marrow microenvironment (Xu et al., 2018).

**TABLE 2:** EVs cargos involved in aging.

Cargos	Functions	References
miRNA	Inhibit BMSCs osteogenic differentiation and induced senescence	Davis et al., 2017
	Promote the oligodendrocyte precursor cells differentiation and improve the ability of remyelination	Pusic and Kraig, 2014
	Promote osteoclast formation and bone reabsorption, leading to the occurrence of osteoporosis	Xu et al., 2018
	Stimulate receptor cell migration and angiogenesis, preventing senescence	van Balkom et al., 2013
	Sensitivity and specificity to predict AD	Lugli et al., 2015
	A novel candidate aging biomarker	Machida et al., 2015; Bertoldi et al., 2018
Inflammatory cargos	Participate in the spread of inflammatory diseases	Boilard et al., 2010; Gomes de Andrade et al., 2018
Nicotinamide phosphoribosyltransferase (eNAMPT)	Promote the biosynthesis of systemic NAD^+^ and offsets the occurrence of aging	Yoshida et al., 2019
DNA	Maintain cellular homeostasis	Ostrowski et al., 2010; Baietti et al., 2012; Takahashi et al., 2017
C24:1 ceramide	A key factor in cell death and senescence	Venable et al., 1995
	Induce senescence of bone marrow mesenchymal stem cells	Khayrullin et al., 2019

*EVs: Extracellular vesicles. BMSCs: Bone marrow mesenchymal stem cells.*

However, exosomes can also suppress cellular senescence in certain contexts. Exosomes have been considered to be messengers of intercellular communication during angiogenesis. Recent studies have revealed that exosomes containing miR-214, produced by the human microvascular endothelial cell line (HMEC-1), can stimulate receptor cell migration and angiogenesis, thereby preventing the development of senescence. In contrast, the depletion of exosome miR-214 in endothelial cells failed to stimulate these processes and prevent cellular senescence (van Balkom et al., 2013). The study of miRNA in young and senescent cell EVs is helpful to reveal the new mechanisms of senescence.

Exosome miRNAs are also considered to be potential attractive biomarkers of aging (Smith et al., 2015). In a study investigating Alzheimer’s disease (AD), Lugli et al. (2015) strongly suggested that plasma exosome miR-1306-5p, which targeted ADAM10, had the best sensitivity and specificity to predict AD, of all indicators examined. In salivary exosomes, miR-24-3p has been identified as a novel candidate biomarker for aging (Machida et al., 2015). The miR-183 cluster of exosomes, comprising miR-96, miR-182, and miR-183, increased during the aging process (Bertoldi et al., 2018). This suggests that the miR-183 clusters have the potential serve as biomarkers of aging.

## Inflammatory Cargos

Inflammation has long been considered a defensive response to microbial agents. It is now clear that an inflammatory response can occur in the absence of infection, in a condition known as “aseptic inflammation” (Chen and Nunez, 2010). Aseptic inflammation is referred to as a chronic systemic inflammatory state during aging (Franceschi et al., 2000), in which EVs are involved (Table 2). EVs can trigger aseptic inflammatory responses by carrying pathogen autoantigens or damage-associated molecular patterns, and are involved in the transmission of inflammatory diseases through EV-associated cytokines, miRNAs, and lipid mediators (Buzas et al., 2014). Boilard et al. (2010) suggested that MPs originating from platelet are able to promote inflammatory reactions and stimulate cytokine responses in synovial fibroblasts via interleukin-1 (IL-1) signaling. Gomes de Andrade et al. (2018) revealed that aging could cause changes in the profiles of circulating exosomes. An age-related increase in CD63 levels was observed in exosomes from cerebrospinal fluid (CSF), and a significant decrease in IL-1β levels was observed in exosomes from the plasma of the older group of male Wistar rats (Gomes de Andrade et al., 2018). These results suggest that changes in IL-1β levels of the exosomes are significantly correlated with age-related inflammatory responses.

## Nicotinamide Phosphoribosyltransferase

Nicotinamide adenine dinucleotide (NAD) is the basic chemical involved in energy metabolism in all living organisms. The expression of NAD^+^, the oxidized form of NAD, in worms, various rodent tissues (fat, skeletal muscle, liver, pancreas, kidney, brain and heart), skin, and neurosensory retina is decreased with age (Braidy et al., 2011; Gomes et al., 2013; Mouchiroud et al., 2013; Khan et al., 2014; Canto et al., 2015; Verdin, 2015; Lin et al., 2018; Rajman et al., 2018; Yoshino et al., 2018). eNAMPT is an essential NAD^+^ biosynthetic enzyme in mammals. In recent years, NAD^+^ metabolism has become a hot topic in the field of aging (Canto et al., 2015; Rajman et al., 2018). Through the enrichment of several exosome markers, such as Flotillin-1, TSG101, CD9, CD63, and CD81, as well as the use of electron microscopy, Yoshida et al. (2019) demonstrated that both mouse and human plasma exosomes contained eNAMPT, which was internalized into target cells to directly enhance cellular NAD^+^ biosynthesis. Moreover, in aging mouse plasma, exosome-containing eNAMPT content can be changed. This is a novel inter-organizational communication mechanism that maintains NAD^+^ levels through exosome-mediated eNAMPT transport. eNAMPT promotes the biosynthesis of systemic NAD^+^, offsets the occurrence of aging, and can be used as a new potential anti-aging intervention pathway (Table 2).

## DNA

EVs also contain chromosomal DNA fragments. It has been reported that no matter the cause of cellular senescence, the secretion of DNA-containing exosomes increases with cell senescence (Takahashi et al., 2017). In senescent human cells, the inhibition of exosome-associated DNA secretion by knocking down Alix or Rab27, which are important molecules for the biogenesis (Baietti et al., 2012) and secretion (Ostrowski et al., 2010) of the exosomes, can provoke reactive oxygen species (ROS)-dependent DNA damage due to the accumulation of DNA in the cytoplasm and senescent cell cycle arrest or cell apoptosis. Even in non-senescent cells, the accumulation of cytoplasmic DNA can induce apoptosis. Cytoplasmic DNA has been reported as a danger signal that activates the innate immune response, including the interferon (IFN) pathway (Abe et al., 2013; Hartlova et al., 2015) and cGAS-STING-dependent signaling (Takahashi et al., 2017). Meanwhile, exosomes are actively secreted from cells in order to the remove infected adenoviral DNA (Takahashi et al., 2017). Exosomes, are therefore believed to play a critical role in senescence-associated secretory phenotypes. These results suggest that exosome secretion can maintain cellular homeostasis by removing harmful cytoplasmic DNA in senescent and non-senescent cells (Table 2). These findings will provide new insights into the control of cell homeostasis as well as new facets to investigate the involvement of EVs.

## C24:1 Ceramide

Ceramide is a sphingolipid produced by the hydrolysis of sphingomyelin, catalyzed by sphingomyelinase (Wang et al., 2012), and has various of forms, such as short-, medium-, long-, and very long-chain. EVs are highly abundant in sphingolipid ceramide (Wang et al., 2012). Lipidomic analyses of serum exosomes indicated that serum exosomes from older women were highly enriched in C24:1 ceramide (Khayrullin et al., 2019). Exosome–associated ceramide has emerged as a key factor in cell death and senescence in a variety of cell types (Venable et al., 1995). Recent studies by Law et al. (2018) revealed that very long-chain ceramides with lipotoxicity can cause mitochondrial dysfunction, oxidative stress, and cell death in cardiomyocytes. *In vitro* experiments have shown that exosomes containing C24: 1 ceramide in serum could induce the senescence of BMSCs (Khayrullin et al., 2019). These results confirm that exosomes containing C24: 1 ceramide may directly lead to the involuntary senescence and apoptosis of cells (Table 2).

These animal and human studies provide strong evidence for the relationship between EVs, cargos, and aging. In the future studies, the relationship between other contents of EVs and senescence should be further explored to reveal the potentially unexpected role of EVs.

## Extracellular Vesicles and Reproductive Diseases

It is widely accepted that normal reproductive processes, including ovulation, menstruation, implantation, and parturition, show signs of inflammation (Goswami et al., 2008). The female reproductive tract needs to solve these problems quickly to restore normal reproductive function. The dysregulation of inflammatory factors play a pivotal role in reproductive diseases (Gupta et al., 2008). As we know, EVs are involved in inflammatory state. In this review, we emphasize the involvement of EVs in PCOS and endometriosis. In view of the functions and characteristics of EVs, we focus on assessing the pathogenic role as well as diagnostic and therapeutic value of EVs in PCOS and endometriosis (Table 3).

**TABLE 3:** Pathogenic role and main functions of EVs in polycystic ovary syndrome and endometriosis.

Disease	Pathogenic role of EVs	Potential functions	References
PCOS	Enhance inflammation and disrupt steroidogenesis by activating the NF-κB signaling pathway		Li et al., 2019
	Alleviate PCOS by targeting PDCD4 to promote proliferation and inhibit apoptosis of cumulus cells	New therapeutic strategy	Zhao et al., 2019
		Biomarkers for predicting PCOS	Carvalho et al., 2017b
Endometriosis	Reflect the state of the inflammation and/or procoagulant system		Munros et al., 2017
	Increase total weight and impair macrophages’ phagocytic ability		Sun et al., 2019
	Contribute to endometriosis by affecting inflammation, angiogenesis, and proliferation in the microenvironment of endometriotic lesions		Khalaj et al., 2019
		Prediction of successful embryo implantation	Parks et al., 2018
	Decrease of endometriosis fibrosis by reducing Collagen αI and CTGF mRNA expression		Wu et al., 2018
	Promote the occurrence of endometriosis and regulate angiogenesis		Harp et al., 2016, Qiu et al., 2019
		A non-invasive detection marker in the diagnosis of endometriosis	Muth et al., 2015

*EVs: Extracellular vesicles. PCOS: Polycystic ovary syndrome. NF-κB: nuclear factor kappa B.*

## Extracellular Vesicles and Polycystic Ovary Syndrome

PCOS is one of the most prevalent endocrine diseases in women of reproductive age (Bozdag et al., 2016). Importantly, women with PCOS have an imbalance between procoagulant and anticoagulant factors with increased levels of proinflammatory cytokines (Repaci et al., 2011; Palomba et al., 2015; Nehir Aytan et al., 2016), which increase the risk of cardiovascular disease. Mesri and Altieri (1998) first described the role of circulating EVs in the spread of endothelial proinflammatory cascades in 1998. They demonstrated that EVs released from polymorphonuclear leukocytes by healthy volunteers could induce endothelial cells to produce cytokines and chemokines *in vitro*. In follicular fluids of PCOS women, Li et al. (2019) used a tandem mass tag quantitative proteomic approach and found that follicular fluid exosomes of both normal and PCOS samples contained S100 calcium-binding protein A9 (S100-A9). S100-A9 expression was greater in the follicular fluid exosomes of PCOS women and enhanced inflammation and disrupted steroidogenesis by activating the nuclear factor kappa B (NF-κB) signaling pathway. This activation was performed in a S100-A9 dose-dependent manner. In the follicular fluids of PCOS patients, EVs miR-132 and miR-320 are expressed at significantly lower levels than in healthy individuals (Sang et al., 2013). Moreover, these two miRNAs are also related to the production of steroidogenesis. However, the women with PCOS, miR-323-3p derived from mesenchymal stem cell exosomes alleviates PCOS by targeting PDCD4 to promote proliferation and inhibit apoptosis of cumulus cells (Zhao et al., 2019). This provides a novel therapeutic strategy for the treatment of PCOS.

MPs are also a type of EVs and play a fundamental role in the communication between source and target cells. Serum MPs’ content can increase in variety of conditions, including PCOS (Willis et al., 2014; Carvalho et al., 2017b), prothrombotic states (Mooberry et al., 2016), and type 2 diabetes mellitus (Koga et al., 2005). Younger women with PCOS have increased concentrations of circulating annexin V-positive platelet MPs in plasma, compared with older women with PCOS (Willis et al., 2014). Surprisingly, in overweight women with the PCOS, plasma MPs are also higher than those in BMI-matched controls (Koiou et al., 2013). It was subsequently confirmed that elevated levels of EVs in women with PCOS are directly related to follicular counts and insulin resistance markers in the ovary (Simon et al., 2018). Interestingly, recent studies have revealed that metformin could reduce total MPs and tissue factor MPs (TFMPs) in women with PCOS (Carvalho et al., 2017a). TFMPs participate in thrombus formation and clot propagation (Nomura and Shimizu, 2015). Moreover, following weight loss in a patient with PCOS, the number of MPs was significantly decreased. The relationship between MPs and the other symptoms of PCOS have not yet been established (Teede et al., 2010).

MPs may serve as useful biomarkers for predicting many diseases, including PCOS and cardiovascular disease (Carvalho et al., 2017b). The ratio of ornithine to arginine in plasma derived from PCOS patients is significantly increased (Kyselova et al., 2019). Furthermore, platelet-derived MPs are the main sources of plasma arginase (Kyselova et al., 2019). Arginase and the ratio of ornithine to arginine have also been reported to represent early biomarkers of potential cardiovascular disease in PCOS patients (Kyselova et al., 2019). Therefore, MPs can be used as potential biomarkers for disease assessment. The role of EVs in PCOS requires further study.

## Extracellular Vesicles and Endometriosis

Endometriosis is a hormone-dependent, chronic, painful, and benign gynecological disorder. Although the pathogenesis of endometriosis is still unknown, Sampson’s can explain the etiology of this disease through the theory of retrograde menstruation. This theory suggests that menstrual discharge returns to the peritoneal cavity through uterine contraction, adheres to the peritoneal tissue, and develops into ectopic lesions. However, of all patients with retrograde menstruation, only 5%–10% develop endometriosis (Cramer and Missmer, 2002; Giudice, 2010). Therefore, endometriosis is considered to be a dysfunctional immune response (Paul Dmowski and Braun, 2004) which promotes inflammatory responses and angiogenesis. Lately, the immune mechanism of endometriosis has received increasing attention (Ballester et al., 2011), notably due to the prevalence of EVs-related endometriosis research. Khalaj et al. (2019) suggested that in endometriosis patients, EVs could contribute to endometriosis by affecting inflammation, angiogenesis, and proliferation in the microenvironment of endometriotic lesions. Sun et al. (2019) reported that endometriosis, an inflammatory disease, possibly involves peritoneal macrophages. EVs derived from eutopic stromal cells of endometriosis could increase the total weight of mice and impair phagocytic ability of macrophages. These stromal cell-derived EVs contribute to the development of lesions. Previous studies by Munros et al. (2017) showed that in deep-infiltrating endometriosis patients, increased levels of circulating total cell-derived MPs may be reflect the state of the inflammation and procoagulant system. The increase in EVs level induces the disorder of immune function, and thus promotes the occurrence of endometriosis. Therefore, EVs have been shown to have the potential to promote endometriosis.

On the other hand, in terms of pathology, the main feature of endometriosis is ectopic tissue fibrosis, which is characterized by excessive deposition of endometriotic tissue on the extracellular matrix (Matsuzaki and Darcha, 2014; Malutan et al., 2015) and may cause scar formation or alter tissue function (Matsuzaki et al., 2010). Wu et al. (2018) reported that the increase in exosome miR-214 from endometrial stromal cells could result in the decrease of endometriosis fibrosis by reducing collagen αI and CTGF mRNA expression. Subsequently, Harp et al. (2016) proposed that exosomes released by endometriotic stromal cells could deliver specific miRNA molecules in an autocrine and paracrine manner to promote the occurrence of endometriosis and regulate angiogenesis. More recently, a study by Qiu et al. (2019) revealed that the serum exosome long non-coding RNA (lncRNA) hypoxia-inducible factor from patients with endometriosis could also promote angiogenesis. These results indicate that exosomes can promote the occurrence of endometriosis and may become an important pathogenesis for endometriosis.

Currently, the combination of laparoscopic evaluation and biopsy is the gold standard for the diagnosis of endometriosis (Gordts et al., 2015). However, both detection methods are invasive. EVs, as non-invasive detection markers, have shown to be value prospective for the diagnosis of endometriosis. Muth et al. (2015) showed that EVs from cervicovaginal lavage and vaginal swabs could be used as a novel and relatively non-invasive method to diagnose primate endometrial disease and other reproductive tract diseases.

## Extracellular Vesicles Application in Clinical and Basic Research

The clinical application of EVs is based on four aspects (Table 4). First, treatment tools. EVs can increase the secretion of proinflammatory cytokines (Prado et al., 2008), and thus reducing the production or absorption of EVs may be a new strategy for the treatment of diseases. Secondly, EVs are promising biomarkers for diagnostic diseases (Lugli et al., 2015; Machida et al., 2015; Muth et al., 2015; Carvalho et al., 2017b; Bertoldi et al., 2018; Kyselova et al., 2019). EVs from bodily fluid have gained significant interest as a potential diagnostic biomarker for various diseases. Thirdly, EVs can be utilized as drug delivery tools. EVs can transport cargos to adjacent cells and be internalized into cells (Yoshida et al., 2019). Lastly, EVs may be applied to vaccinations. EVs have the potential to improve immune function (Raposo et al., 1996; Zitvogel et al., 1998; Chaput and Thery, 2011). The properties of EVs regulate the immune system, and thus give them the possibility to be involved in vaccinations.

**TABLE 4:** EVs application in clinical and basic research.

EVs application	References
**Clinical**	
Treat tools	Prado et al., 2008
Promising biomarkers for diagnostic diseases	Lugli et al., 2015; Machida et al., 2015; Muth et al., 2015; Carvalho et al., 2017b; Bertoldi et al., 2018; Kyselova et al., 2019
Drug delivery tools	Yoshida et al., 2019
Vaccines	Raposo et al., 1996; Zitvogel et al., 1998; Chaput and Thery, 2011
**Basic research**	
Function: Influence inflammation, angiogenesis, disrupting steroidogenesis and impairing macrophages’ phagocytic ability. Alleviation diseases development.	Mesri and Altieri, 1998; Boilard et al., 2010; Harp et al., 2016; Munros et al., 2017; Gomes de Andrade et al., 2018; Khalaj et al., 2019; Li et al., 2019; Qiu et al., 2019; Sun et al., 2019
Mechanism: the NF-κB signaling pathway	Li et al., 2019; Zhang et al., 2019
	Wu et al., 2018; Liu et al., 2019; Mobarak et al., 2019; Yoshida et al., 2019; Zhao et al., 2019

*EVs: Extracellular vesicles. NF-κB: nuclear factor kappa B.*

In basic research, on the one hand, EVs contribute to disease pathophysiology. EVs and cargos include protein, miRNAs and lncRNAs that promote the development of the disease by influencing inflammation, angiogenesis, steroidogenesis and macrophage phagocytic ability (Mesri and Altieri, 1998; Boilard et al., 2010; Harp et al., 2016; Munros et al., 2017; Gomes de Andrade et al., 2018; Khalaj et al., 2019; Li et al., 2019; Qiu et al., 2019; Sun et al., 2019). This effect may be mediated by the NF-κB signaling pathway (Li et al., 2019; Zhang et al., 2019). On the other hand, EVs can also alleviate disease development (Wu et al., 2018; Liu et al., 2019; Mobarak et al., 2019; Yoshida et al., 2019; Zhao et al., 2019). It may also be a new pathway to treatment diseases.

## Conclusion

In the recent decades, research on aging and reproductive diseases is mainly based on traditional fields such as genetic alterations and epigenetics. For this reason, many studies have focused on the development of EVs. Advances in the understanding of EVs in recent years have provided remarkable revelations. The relationship between EVs and aging as well as reproductive diseases has attention widespread attention. EVs could have the potential to be a new and non-invasive marker for assessing the condition of aging and reproductive diseases. In-depth research on the role and mechanism of EVs will provide new strategies for delaying aging and treating reproductive diseases and may eventually provide major innovations in their diagnosis and treatment. While EVs have shown potential importance and significance, stronger evidence is needed to support the possibility of EVs as clinical application. In addition, the lack of a gold standard method for EV separation and purification is a challenge and will limit the possibilities and significance of subsequent functional research.

## Author Contributions

YL carried out literature search, data collection and analysis, and wrote the manuscript. QS revised the manuscript. LZ carried out design and revised the manuscript. WX took part in design and revised the manuscript. All authors read and approved the manuscript.

## Conflict of Interest

The authors declare that the research was conducted in the absence of any commercial or financial relationships that could be construed as a potential conflict of interest.
